# Understanding freelancers: optimizing HRM for freelancer success

**DOI:** 10.3389/fpsyg.2026.1722270

**Published:** 2026-04-28

**Authors:** Edna Rabenu, Yonatan Shertzer, Aharon Tziner, Daphna Shwartz-Asher, Yahel Kurlander, Sari Ehrlich, Yossef Tobol, Hilla Peretz

**Affiliations:** 1 Human Services Department, Tel-Hai Academic College, Upper Galilee, Israel; 2 Human Resources Department, Peres Academic Center, Rehovot, Israel; 3 Organizational Behavior Department, Tel-Hai Academic College, Upper Galilee, Israel; 4 Economics and Management Department, Tel-Hai Academic College, Upper Galilee, Israel; 5 Industrial Engineering and Management Department, Braude College of Engineering, Karmiel, Israel

**Keywords:** freelancers, HR practices, knowledge sharing, performance, social exchange theory (SET)

## Abstract

**Introduction:**

The rise in non-traditional employment has reshaped the labor market, with freelancers emerging as a significant and increasingly strategic segment of the workforce. Freelancers are highly skilled professionals who contribute to knowledge-intensive and innovative projects, yet despite their growing importance, they are often overlooked by HR practitioners and excluded from formal HR processes. This study examines how HR practices affect organizational perceptions of freelancers with particular attention to the instrumental mechanisms through which HR practices shape managerial perceptions of freelancers, especially performance and knowledge sharing.

**Method:**

Data was collected in two waves from 468 managers and employees working in organizations that employ independent contractors.

**Results:**

Results indicate that performance and knowledge sharing provide the clearest pathways linking HR practices to positive managerial perceptions of freelancers, whereas relational mechanisms received limited support.

**Discussion:**

The findings support a freelancer-specific HRM model, in which instrumental mechanisms are more central than employee-like relational exchange processes.

## Introduction

In the past two decades, we have witnessed a rise in Western economies in contracting with workers operating as freelancers moving from one project to the next (Barlage et al., 2023). Non-traditional employment methods such as freelancing are vastly different from the traditional employment model (Zadik et al., 2019; Santra, 2021). Indeed, freelancing, once a niche concept, has become a mainstream career choice for many (Lo Presti et al., 2018). This shift is driven by various factors, including technological advancements that facilitate remote work, the desire for greater flexibility and autonomy, and the need for organizations to access specialized skills on a project basis (Akhmetshin et al., 2018). As a result, organizations are increasingly turning to freelancers to supplement their workforce and address evolving business needs (Lo Presti et al., 2018).

While contracting freelancers offers organizations numerous benefits such as access to specialized talent (Akhmetshin et al., 2018) and offers the freelancers autonomy and flexibility (Spurk and Straub, 2020), their employment, however, presents unique challenges. One such challenge is integrating the freelancers into the organization’s culture and the workflows (Rabenu, 2021), a challenge that is facing the Human Resources department. If we accept that HR is ‘the process through which management builds the workforce and tries to create the human performance that the organization needs’ (Boxall and Purcell, 2022), then regarding freelancers, there is a gap. Although they are part of that workforce within the ecosystem, freelancers are currently overlooked or ignored by HR practice and research (Cross and Swart, 2022). Indeed, freelancers often do not encounter HR personnel, nor are they part of the HR organizational practices; rather, they are directly contracted with managers or the firm’s financial department.

Freelancers’ “neglect” by HR can result in significant negative deleterious effects on individual well-being (Vučeković et al., 2023; Jacobs et al., 2019) and the overall experience of work and life, but perhaps more significantly, this disregard can have a substantial impact on organizational outcomes and performance (Barney, 2018). Because freelancers are engaged primarily through transactional contracts, rather than through standard HR practices, they are essentially marginalized with concomitant negative ramifications on their employment (Butterick and Charlwood, 2021). To maximize the potential of this workforce model, organizations should examine the strategies they employ to foster engagement, collaboration, and performance (Barlage et al., 2023).

Despite being external to formal employment systems, freelancers increasingly take part in knowledge-intensive, collaborative, and strategically important organizational work (Shwartz-Asher et al., 2025; Zadik et al., 2019). When freelancers contribute to tasks that require coordination with internal employees, shared information, or alignment with organizational expectations, organizations face a practical tension: freelancers must remain legally and structurally independent, yet their effective contribution often depends on processes traditionally associated with HRM, such as training, feedback, or communication routines (McKeown and Pichault, 2021). Thus, HR involvement becomes relevant not as an attempt to internalize freelancers, but as a way to support collaboration and ensure that project-based work meets organizational needs. This tension underscores the importance of understanding when and how specific HR practices shape freelancers’ behaviors and influence managers’ perceptions of their value.

Although freelancers now represent a growing and critical share of the workforce, only a handful of pioneering studies have examined the gap in HR’s treatment of freelancers compared to traditional employees. Prior conceptual models, such as the UPTOP (Unified Pool of Talents in Organization Project-array) framework (Rabenu, 2021), suggest that narrowing this gap could enhance freelancers’ integration and promote positive outcomes such as stronger engagement, greater knowledge sharing, and increased perceptions of job security (see also Zadik et al., 2019). Yet empirical research has not systematically tested these claims, leaving open questions about how HR practices actually shape freelancers’ behaviors and, ultimately, organizational perceptions of their value.

Even though freelancers operate within multi-actor ecosystems and frequently maintain portfolio careers across several clients, organizations remain a central actor in shaping freelancers’ work experiences. Recent research on inclusive HRM (van den Groenendaal et al., 2023) shows that organizations selectively extend HR practices to external contributors when coordination, knowledge flow, and project alignment are essential. In this sense, HRM is not conceptualized as a mechanism tied to traditional employment stability, but rather as a set of practices that organizations may deploy strategically to support collaboration with independent professionals. Although freelancers may simultaneously respond to expectations originating from multiple clients or institutional environments, our model examines how managers in the contracting organization perceive freelancers’ behaviors shaped by HR practices.

**Figure 1:**
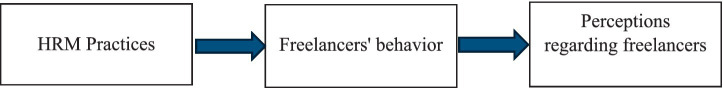
Research model.

The present study directly addresses this gap by exploratorily investigating the association between applied HR practices and attitudes toward hiring freelancers, focusing on the mediating role of freelancers’ behaviors. Given the limited empirical research on HRM and freelancers, the present study adopts an exploratory approach to examine which mechanisms are most likely to operate in freelance contexts, with particular emphasis on instrumental pathways. By focusing on these mechanisms, the study might offer a more precise understanding of how HR practices shape freelancers’ outcomes and managerial perceptions (see Figure 1).

We call upon Zadik et al.'s (2019) call for future investigations of how managers in organizations that employ freelancers perceive this growing phenomenon, and under which circumstances this type of employment will be most beneficial for the organizations. Additionally, we answer van den Groenendaal et al.'s (2023) appeal to investigate further the role of HRM practices in successful freelancers’ employment. Finally, we respond to Cross and Swart’s (2022) insistence that there be a more refined and nuanced HR approach that encompasses the complexity of freelancers’ employment.

### Freelancers’ work

In recent years, employment arrangements have changed from regular salaried work to alternative plans such as independent contracting or freelancing. Based on Rabenu’s (2021, p. 227) definition of freelancers as “self-employed workers, mostly professionals and experts, who work by themselves under a bounded time/project-based contract, agreed directly between them and the client,” freelancers are not employees: They are independent contractors who enter into direct contracts with clients who define and outline work results. Moreover, freelancers may work for several clients simultaneously and are usually employed on a temporary project basis (Kitching and Smallbone, 2012).

In 2019, the percentage of self-employed workers in the UK was more than 15.3% (ONS, 2018), in the EU, 15.3%, in Japan 10%, and in Korea 24.6% (OECD, 2021). Notably, organizations are more likely than ever to utilize project-based temporary expertise hiring, taking advantage of specialized skills provided by valuable and scarce knowledge workers rather than the generic and lower-value skills characteristic of the 20th century (Barlage et al., 2023).

The traditional employment of freelancers prevalent in the 20^th^ century was thought to offer businesses competitive and effective employment options, particularly during recessions (Zadik et al., 2019). However, the more recent practice of freelance employment that has become more common in the past two decades is a new class of talented independent contractors, known as “Super temps” (Miller and Miller, 2012). These talented individuals are highly qualified and specialized human capital workers, who sometimes even earn more than comparable full-time workers. Burke (2015) indicates that these top freelancers work on projects requiring strategy, innovation, and technological advancement and are not cost-effective or a short-term substitute for full-time workers. As Zadik et al. (2019) contend: The freelancers are hired by the company because of their unique skills and knowledge and not as a means for cost-reduction.

At the same time, there are several shades of freelancers with many nuances to consider, such as contract duration (long/short term period), whether freelancing is a primary or secondary job, the worker’s position in the labor market, and the nature of the relationship between freelancer and client (Kitching and Smallbone, 2012; Poon, 2019; Rabenu, 2021, p. 227). As such, for example, freelancers under short-term contracts rarely anchor their careers within a single organization; rather, they operate across fluid ecosystems of clients, platforms, and professional networks (Kitching and Smallbone, 2012; Lo Presti et al., 2018). This multi-actor environment shapes their autonomy and identity and differentiates them from traditional employees. Acknowledging this broader context, our analysis focuses specifically on practices enacted by the contracting organization, recognizing that these practices represent only one, yet a highly relevant, set of influences within the freelancer’s broader career system.

### Human resource management and freelancers

From an organizational standpoint, freelancers are not employees. Consequently, they are not generally subject to reciprocal obligations extant in standard employment relationships or bound by an organization’s directive control (Spreitzer et al., 2017). This assumption leads to a gap in the policy and procedures concerning HR practices and freelancers’ employment. Indeed, HR is often excluded from decisions regarding outsourcing human capital (Camuffo and De Stefano, 2019). Furthermore, when it comes to freelancers, HR departments typically do not influence decisions or offer the services they provide to regular (internal) employees, such as selection, training, special benefits, and participation in social events (Cross and Swart, 2022). In contrast, most decisions concerning freelancers’ employment are made *ad hoc* or at the discretion of individual managers. Rather than being a reflection of a deliberate decision, the exclusion of HR signifies the transactional nature of the relationship (Leighton and Wynn, 2011): Freelancers are viewed as a commodity in the market rather than as human beings (Butterick and Charlwood, 2021). On the individual level, such a perception may cause freelancers to undergo an inherent stress resulting in feelings of neglect and invisibility (Spreitzer et al., 2017) with substantial ramifications on their well-being and health (Butterick and Charlwood, 2021).

Furthermore, there is a substantial legal ambiguity related to hiring freelancers. To prevent possible legal issues and the complexities of compensation, organizations attempt to maintain a clear distinction and separation between the status of independent contractors and employees (Adams et al., 2018). They do so by providing freelancers with an explicit legal contract that specifies the relationship, exact scope of work, and the precise boundaries of the project, explicitly stating the absence of any benefits and perks that could identify them as employees (Snider, 2018). Because the organization deliberately distinguishes between the freelancer and salary-based employees, creating insiders and outsiders, this legal ambiguity has socio-cultural consequences (Cross and Swart, 2022).

Thus far, research on HRM strategies for independent contractors, such as the HR Architecture Model (Lepak and Snell, 1999) and the Flexible Firm Model (Atkinson, 1984), has [also] tended to primarily view freelancers as an organization’s periphery with little access to HRM (Beer et al., 2015). In these models, organizations are advised to invest in core employees through HRM and to provide only the most essential or basic HRM activities to the periphery, including freelancers (Lepak and Snell, 1999). However, as indicated, the freelancers’ standing in the labor market and, consequently, their long-term careers may suffer due to this exclusive HRM approach (De Vos and Van der Heijden, 2017). Importantly, our conceptual model does not assume that freelancers engage in linear, employee-like relational exchanges with the organization or that their work resembles stable traditional employment. Instead, we adopt a targeted HR-architecture perspective that asks a narrower question: when organizations *do* apply selected HR practices to freelancers, how do these practices influence freelancers’ behaviors and, ultimately, managers’ perceptions? Prior studies show that even project-based contractors respond to organizational cues such as access to strategic information, feedback, and training (Fong et al., 2011).

Moreover, Koriat and Gelbard (2014) found that the type of employment contract is a central factor shaping how workers perceive human resource practices (HRP), perceived organizational support (POS), Means and External efficacy and identification with the client organization. This set of mechanisms, organized as an integrative model converges to enhance collaborative behaviors, particularly knowledge sharing. Accordingly, different patterns of collaboration can be expected between employment statuses: internal versus external workers (freelancers in the current research).

Therefore, examining organizational HR touchpoints is theoretically meaningful even if freelancers simultaneously interact with multiple clients or maintain portfolio careers. Our model isolates the organizationally controlled practices from this broader ecosystem to understand their specific effects on perceptions and outcomes.

### HRM practices and freelancers

HRM practices are a collection of actions designed to improve an organization by managing its human resources. They encompass various activities such as resourcing, learning and development, performance and reward management, employee relations, and administration (Boxall and Purcell, 2022).

Studies have shown that satisfaction with HRM practices has been associated with performance (Stirpe et al., 2022), engagement (Memon et al., 2021), Organization Citizenship Behavior (OCB) (Taamneh et al., 2018; Gong, 2025), Knowledge sharing (Fong et al., 2011), Counterproductive Work Behavior (CWB) (Carpenter et al., 2021), and more. Consequently, if organizational management wishes to apply these HR services to freelancers, they must devise a comprehensive strategy that encompasses this swiftly expanding trend while accounting for the intricate legal, policy, and socio-cultural aspects of freelance contracting. Specifically, we suggest a model that examines the association between applied HR practices and perceptions towards hiring freelancers, mediated by freelancers’ behaviors and outcomes.

Social Exchange Theory (SET) provides a useful framework for understanding how HR practices may shape freelancers’ behaviors. However, its application to freelance work is not straightforward. Unlike traditional employees, freelancers are not embedded in long-term organizational relationships and are not necessarily expected to engage in ongoing reciprocal exchanges.

Accordingly, we distinguish between two types of exchange mechanisms. Relational mechanisms (e.g., engagement and OCB) rely on identification, mutual commitment, and repeated interactions, which are less characteristic of freelance arrangements. In contrast, instrumental mechanisms (e.g., performance and knowledge sharing) are more consistent with the project-based and contractual nature of freelance work. Accordingly, rather than assuming that all SET-based mechanisms operate equally, the present model examines their differential relevance in freelance contexts.

Unbalanced expectations between freelancers and the organization may lead to a psychological contract breach, which is a subjective experience that refers to the perception by one of the parties that the other has failed to sufficiently fulfill their obligations and commitments (Topa et al., 2022). This breach has been demonstrated to lead to undesirable emotions, attitudes, and behaviors of the parties involved (Zhao et al., 2007). Therefore, when HR practices applied only to employees and not applied to freelancers, they may feel a psychological contract breach. Indeed, several studies have shown that when the psychological contract is breached, it can result in CWB and a lack of OCB behavior (Griep et al., 2016), which is regarded as emotional relational mechanisms. Conversely, the organization may feel a violation of a psychological contract by the freelancers because, by sticking to the specific confines of their contract, the freelancers generate and perpetuate the tangible exchange. For instance, they will not share information and demonstrate low OCB. These unconstructive behaviors readily translate into organizations’ adverse perceptions regarding their commitment to employ freelancers in the future.

Given the project-based, bounded, and relatively independent nature of freelance work, the present study focuses on the instrumental mechanisms that are most theoretically aligned with this employment form. Specifically, HR practices applied to freelancers are expected to shape managerial perceptions primarily through task-relevant pathways rather than through employee-like relational attachment mechanisms. Hence, we hypothesize:

*H1*: The relationship between HR practices applied to freelancers and managerial perceptions of freelancer employment will be positively mediated by knowledge sharing.

*H2*: The relationship between HR practices applied to freelancers and managerial perceptions of freelancer employment will be positively mediated by performance.

**Figure 2:**
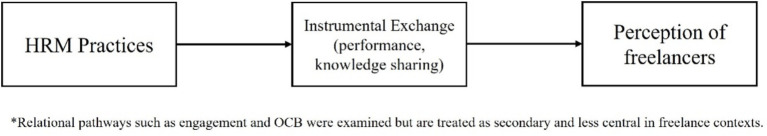
Research model. Relational pathways such as engagement and OCB were examined but are treated as secondary and less central in freelance contexts.

Additional relational pathways (e.g., engagement, OCB, and CWB) were examined on an exploratory basis (Figure 2).

### Methodology

#### Procedure

Data was collected using the Prolific online platform. The study was conducted in three stages. First, a screening phase ensured that participants were relevant: 808 individuals completed a pre-screening survey, and 523 (65%) confirmed that their organization employed freelancers. Only these respondents moved on to the main survey.

In the second stage, the complete questionnaire was administered to the qualified participants, resulting in 468 valid responses (an 89% response rate). In the third stage, five months later, the same participants were contacted again and invited to complete the survey again. A total of 366 individuals completed the second wave, yielding a retention rate of 78%. This allowed us to assess temporal stability and test–retest reliability.

Given the limited research on human resource management (HRM) and freelancers, the study took on a partly exploratory design. The survey was developed by the authors and included both structured items and open-ended questions; however, only the quantitative measures are analyzed in this paper. This study is part of a broader research program focused on the organizational integration of freelancers.

### Participants

The results are based on statistical analyses conducted on the first wave of respondents. This initial sample included 468 participants, comprising 259 men, 207 women, and two non-binary individuals. The respondents were geographically diverse, with 347 residing in Europe, 84 in Africa, 22 in North America, 6 in Asia, 5 in South America, and 4 in Australia. The ages of participants ranged from 20 to over 60, with the majority falling between 20 and 40 years old. They represented various industries, including services (166), hi-tech (75), industry (65), finance (37), healthcare (28), education (20), trade (18), government (10), retail (4), and other fields (45).

#### Measures

From the complete questionnaire, the following variables were selected for the current in the study.

*Perceptions of Freelancer Employment* were evaluated using two questions. The first question asked participants to rate their willingness to recommend freelancers on a scale from 1 to 6, where 1 means “definitely no” and 6 means “definitely yes.” The second question focused on trend perception, asking participants to rate their view of the trend in employing freelancers on a scale from 1 to 5, where 1 indicates “very negative” and 5 indicates “very positive.” These questions reflect the participants’ overall perception of freelancers’ employment. We acknowledge the possible limitations of using single-item measures; however, prior research shows that single-item measures of attitudes can match multi-item scales in predictive validity, convergent validity, and reliability under certain conditions, challenging the assumed superiority of multi-item approaches (e.g., Ang and Eisend, 2018; Bergkvist and Rossiter, 2007). At the same time, given the study’s focus on general evaluative perceptions, concise measures are appropriate and have been shown to capture attitudinal constructs effectively in similar contexts.

*Freelancer Behavior* was evaluated through 23 items comparing freelancers to salaried employees on dimensions such as performance, engagement, organizational citizenship behavior (OCB), and counterproductive work behavior (CWB), using a 5-point comparative scale (1 = Freelancers much less than salaried employees, 5 = Freelancers much more than salaried employees). Importantly, these measures capture respondents’ comparative perceptions of freelancers relative to salaried employees rather than objective behavioral frequencies. As such, they reflect perceived rather than actual behaviors and are interpreted accordingly. This approach is consistent with the study’s focus on managerial evaluations, which are inherently perceptual and shape decision-making regarding freelancer employment. Accordingly, the model examines perceived mechanisms through which HR practices are associated with managerial evaluations of freelancers, rather than directly observed freelancer behaviors.

*Knowledge Sharing* was measured using five items assessing the degree to which freelancers engage in collaborative learning or contribute knowledge to others (e.g., “train the team in their area of expertise”). Participants chose from four options: (1) no difference between freelancers and salaried employees, (2) depends on the freelancer, (3) freelancers do not engage in this behavior, or (4) the item is irrelevant in their organization.

*HRM Practices* were evaluated via nine items that examined how organizations include freelancers in various human resource processes: training and instruction, assessment/feedback, receiving benefits (e.g., food cards, gifts, etc.), team spirit events, exposure to organization’s vision and values, exposure to strategic issues, exposure to internal tenders, access to all the organizational data, work meetings. These were measured using the same response format as knowledge-sharing items. The HRM practices index was calculated as the average of the nine individual practices. The response categories for these items were transformed into numeric values to enable aggregation. Specifically, responses indicating the presence of a practice (e.g., inclusion in training, access to information) were coded as higher values, while responses indicating absence were coded as lower values. The “depends on the freelancer” option was treated as a mid-point reflecting variability in practice application. Responses marked as “irrelevant” were treated as missing and excluded from the analysis. Composite indices were then calculated as the average across available items for each respondent. While this transformation simplifies nuanced responses, it allows for capturing overall patterns of HR practice inclusion.

Construct validity and reliability for all measures were established through factor analyses and are reported below.

## Results

### Measurement validation and descriptive findings

To evaluate the construct validity of the questionnaires used in the study, three exploratory factor analyses (EFAs) with Promax rotation were performed, one for each questionnaire. These analyses were conducted using data from the first wave of data collection (T1). Following this, data from the second wave (T2) was utilized for a confirmatory factor analysis (CFA) of all scales simultaneously. Table 1 summarizes the factor loadings of the measures.

**Table 1:** Post Promax rotation factor loadings of the freelancers’ behavior questionnaire.

Item	OCB	Performance	Emotional affinity and engagement	CWB
Free_behav18	**0.83**	−0.03	−0.12	0.01
Free_behav16	**0.82**	−0.05	−0.15	0.06
Free_behav19	**0.78**	−0.21	0.11	0.03
Free_behav20	**0.73**	0	−0.05	0.13
Free_behav14	**0.71**	0.05	−0.02	−0.03
Free_behav17	**0.70**	−0.01	−0.04	−0.11
Free_behav15	**0.62**	0.14	−0.08	−0.13
Free_behav21	**0.43**	0.13	0.07	0.05
Free_behav9	−0.1	**0.94**	−0.11	0.01
Free_behav11	−0.11	**0.87**	0.05	0.04
Free_behav10	0.04	**0.82**	0.03	0
Free_behav8	0.07	**0.72**	0	−0.04
Free_behav1	−0.15	−0.14	**0.87**	0.01
Free_behav2	−0.08	0.03	**0.81**	−0.03
Free_behav3	−0.14	0.06	**0.78**	0.04
Free_behav4	0.21	−0.06	**0.51**	−0.05
Free_behav6	0.18	0.12	**0.45**	−0.06
Free_behav5	0.27	0.05	**0.45**	0.07
Free_behav22	0.02	0.03	−0.04	**0.92**
Free_behav23	0.03	0.01	0.04	**0.92**

Bold values indicate the highest factor loading for each item and represent the factor on which the item primarily loads.

**Figure 3:**
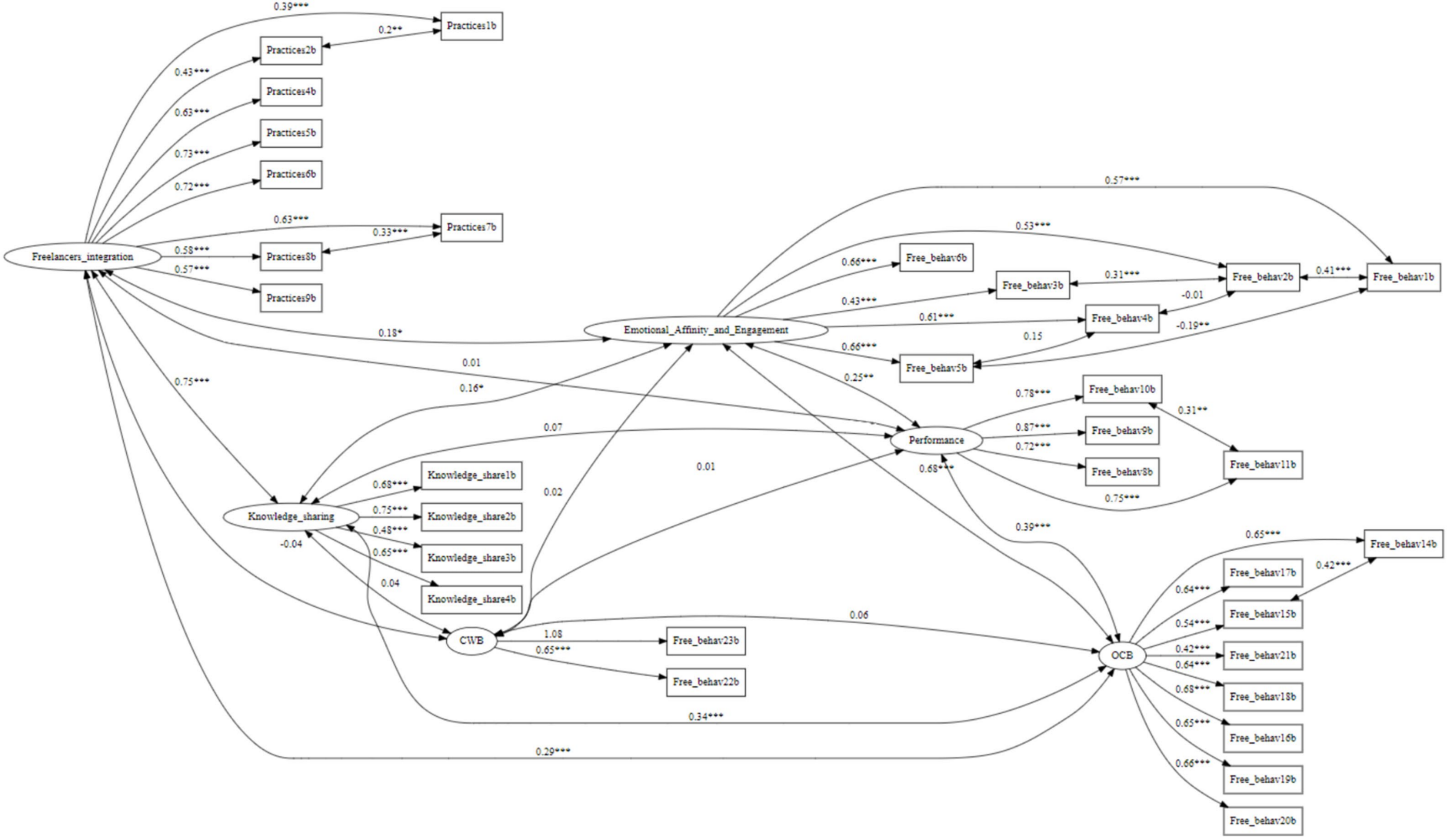
Standardized factor loadings from the CFA. *p < 0.05, **p < 0.01, ***p < 0.001. All data was taken from the second measurement point (T2). Two sided arrows represent correlations while one sided arrows from latent variables to observed variables represent factor loadings. All nine added correlations based on MI suggestions are presented in the figure.

The six-factor measurement model, including *HRM practices, freelancer engagement, organizational citizenship behavior (OCB), counterproductive work behavior (CWB), knowledge sharing, and freelancer performance*, exhibited good fit to the data. Figure 3 presents the standardized factor loadings for the six-factor model.

Confirmatory factor analysis produced acceptable indices (χ^2^ (440) = 843.34, *p* < 0.001; RMSEA = 0.05; SRMR = 0.06; CFI = 0.91), confirming the distinctiveness of the constructs. Reliability was strong across all scales, with Cronbach’s alpha coefficients ranging from 0.74 to 0.86.

Descriptive statistics and bivariate correlations are presented in Table 2.

**Table 2:** Descriptive statistics and Pearson correlation coefficients between the study variables—Time 1, *n* = 466.

Variable	*M*	*SD*	1	2	3	4	5	6	7
1. Willingness to recommend freelancers	4.84	0.89							
2. Attitude toward freelancer employment	3.54	0.96	0.49**						
3. HRM practices index	1.96	0.46	0.07	0.09					
4. Knowledge Sharing	2.19	0.55	0.14**	0.20**	0.42**				
5. OCB	2.43	0.69	0.18**	0.07	0.18**	0.16**			
6. Performance	3.05	0.76	0.19**	0.19**	0.05	0.07	0.42**		
7. Emotional affinity and engagement	1.94	0.62	0.14**	0.09	0.10*	0.10*	0.47**	0.29**	
8. CWB	2.77	0.86	−0.06	−0.09*	0.11*	0.13**	0.08	−0.02	0

*p < 0.05, **p < 0.01.

As expected, HRM practices were positively correlated with knowledge sharing (*r* = 0.42, *p* < 0.01), and OCB (*r* = 0.18, *p* < 0.01), and moderately associated with CWB (*r* = 0.11, *p* < 0.05) and engagement (*r* = 0.10, *p* < 0.05). The willingness to recommend freelancers was positively related to engagement (*r* = 0.14, *p* < 0.01), OCB (*r* = 0.18, *p* < 0.01), performance (*r* = 0.19, *p* < 0.01), and knowledge sharing (*r* = 0.14, *p* < 0.01). Attitudes toward freelancers were positively related to performance (*r* = 0.19, *p* < 0.01), and knowledge sharing (*r* = 0.20, *p* < 0.01).

All comparisons between variables at time 1 and time 2 were tested. Cross-time mean difference and correlation tests (n = 335) were conducted (see Table 3), and no significant difference was found between them. Therefore, we focused on time 1 to examine the hypotheses.

**Table 3:** Descriptive statistics and Pearson correlation coefficients between the study variables—Time 2, *n* = 366.

Variable	*M*	*SD*	1	2	3	4	5	6	7
1. Willingness to recommend freelancers	4.73	0.95							
2. Attitude toward freelancer employment	3.45	0.94	0.49**						
3. HRM practices index	1.96	0.49	0	0.03					
4. Knowledge Sharing	2.19	0.54	0.18**	0.08	0.55**				
5. OCB	2.38	0.67	0.18**	0.13*	0.25**	0.22**			
6. Performance	2.97	0.75	0.27**	0.27**	0.01	0.03	0.41**		
7. Emotional affinity and engagement	1.93	0.62	0.16**	0.12*	0.10	0.04	0.54**	0.30**	
8. CWB	2.84	0.88	−0.02	−0.04	−0.01	0.03	0.06	0.03	0.07

*p < 0.05, **p < 0.01.

### Common method bias

Due to the cross-sectional nature of our study, we took precautions to assess potential common method bias. We applied Harman’s one-factor test, following the procedure outlined by Chang et al. (2010). This approach assumes that if a single unrotated factor accounts for less than 50% of the total variance, common method bias is unlikely to be a significant issue. While the first factor explained 27% of the variance, suggesting that a single dominant factor is not present, this test alone is insufficient to fully rule out common method bias, and the results should therefore be interpreted with caution. Given the reliance on self-reported and single-source data, the potential influence of common method bias cannot be entirely excluded.

### Mediation analyses

To examine the research hypotheses of the relations between HRM practices and attitude toward Freelancer employment, as well as the mediating roles of Knowledge sharing, OCB, Performance, Emotional affinity, engagement, and CWB, a mediation analysis was conducted using Hayes (2012) Process, model 4, an addon to R. Summary of the results appears in Table 4.

**Table 4:** Mediation model coefficients.

Variable	Step 1					Step 2		Step 3	
	Emotional affinity and engagement (β, P) [95% CI]	OCB (β, P) [95% CI]	CWB (β, P) [95% CI	Knowledge sharing (β, P) [95% CI]	Performance (β, P) [95% CI]	Freelancer recommendation (β, P) [95% CI]	Trend in employing freelancers (β, P) [95% CI]	Freelancer recommendation (β, P) [95% CI]	Trend in employing freelancers (β, P) [95% CI]
HRM practices index	(0.10, 0.03) [0.01, 0.26]	(0.18, <0.01) [0.14, 0.41]	(0.11, 0.02) [0.03, 0.36]	(0.42, <0.01) [0.39, 0.59]	(0.05, 0.31) [−0.07, 0.23]	(−0.004, 0.94) [−0.20, 0.19]	(0.02, 0.73) [−0.17, 0.24]		
Emotional affinity and engagement						(0.05, 0.37) [−0.08, 0.22]	(0.02, 0.71) [−0.13, 0.19]		
OCB						(0.10, 0.09) [−0.02, 0.27]	(−0.04, 0.51) [−0.21, 0.10]		
CWB						(−0.09, 0.05) [−0.19, 0.001]	(−0.15, <0.01) [−0.27, −0.07]		
Knowledge sharing						(0.12, 0.02) [0.04, 0.37]	(0.19, <0.01) [0.18, 0.53]		
Performance						(0.12, 0.02) [0.03, 0.27]	(0.20, <0.01) [0.12, 0.37]		
HRM practices via engagement								(−0.00, 0.42) [−0.01, 0.04]	(0.00, 0.73) [−0.02, 0.03]
HRM practices via OCB								(0.02, 0.14) [−0.01, 0.09]	(−0.01, 0.52) [−0.06, 0.03]
HRM practices via CWB								(−0.01, 0.14) [−0.05, 0.00]	(−0.02, 0.06) [−0.07, −0.00]
HRM practices via Knowledge sharing								(0.05, 0.04) [0.01, 0.20]	(0.08, <0.01) [0.08, 0.28]
HRM practices via Performance								(0.01, 0.41) [−0.01, −0.07]	(0.01, 0.37) [−0.02, 0.53]

Step 1 = direct relationship between independent variable to mediators; Step 2 = direct relationship between independent variable and mediators to dependent variables; Step 3 = indirect relationship between independent variable to dependent variables via mediators.

Analysis was conducted in three steps. In step 1, we examine the direct effect of HRM practices index on the mediators (knowledge sharing, OCB, performance, emotional affinity, engagement, and CWB) (see Table 4, step 1). The results indicated that HRM practices index positively relates to knowledge-sharing (*β* = 0.42, *p* < 0.001), OCB (*β* = 0.18, *p* < 0.001), Emotional affinity and engagement (*β* = 0.10, *p* = 0.03), as well as CWB (*β* = 0.11, *p* = 0.02). In step 2, we examine the direct effect on HRM practices index and all the mediators on the dependent variables, willingness to recommend freelancers and trend in employing freelancers. The results (see Table 4, step 2) indicated that HRM Practices index is not related directly to neither willingness to recommend freelancers (*β* = 0.004, *p* = 0.94) nor trend in employing freelancers (*β* = 0.02, *p* = 0.73). Additionally, mediators that were found to be directly related to the dependent variables were knowledge-sharing (*β* = 0.12, *p* < 0.001 for willingness to recommend freelancers and *β* = 0.19, *p* < 0.001 for trend in employing freelancers), Performance *(β* = 0.12, *p* < 0.001 for willingness to recommend freelancers and *β* = 0.20, *p* < 0.001 for trend in employing freelancers), and CWB (*β* = −0.09, *p* = 0.05 for willingness to recommend freelancers and *β* = −0.15, *p* = 0.001 for trend in employing freelancers), whereas OCB (*β* = 0.10, *p* = 0.09 for willingness to recommend freelancers and *β* = −0.04, *p* = 0.51 for trend in employing freelancers), and emotional affinity and engagement (*β* = 0.05, *p* = 0.37 for willingness to recommend freelancers and *β* = 0.02, *p* = 0.71 for trend in employing freelancers) were not significant direct predictors.

In step 3, and in order to confirm the hypotheses, we conducted bootstrap procedure based on 10,000 subsamples (see Table 4, step 3). For the depend variable willingness to recommend freelancers, the results indicated that only the indirect path through Knowledge-sharing (*β* = 0.08, 95%CI [0.08, 0.28], *p* < 0.01) was found to be significant. For trend in employing freelancers, the results indicated that indirect paths through knowledge-sharing (β = 0.05, 95%CI [0.01, 0.20], *p* < 0.05) and CWB (β = −0.03, 95%CI [−0.003, −0.07], *p* = 0.05) were found to be significant.

**Figure 4:**
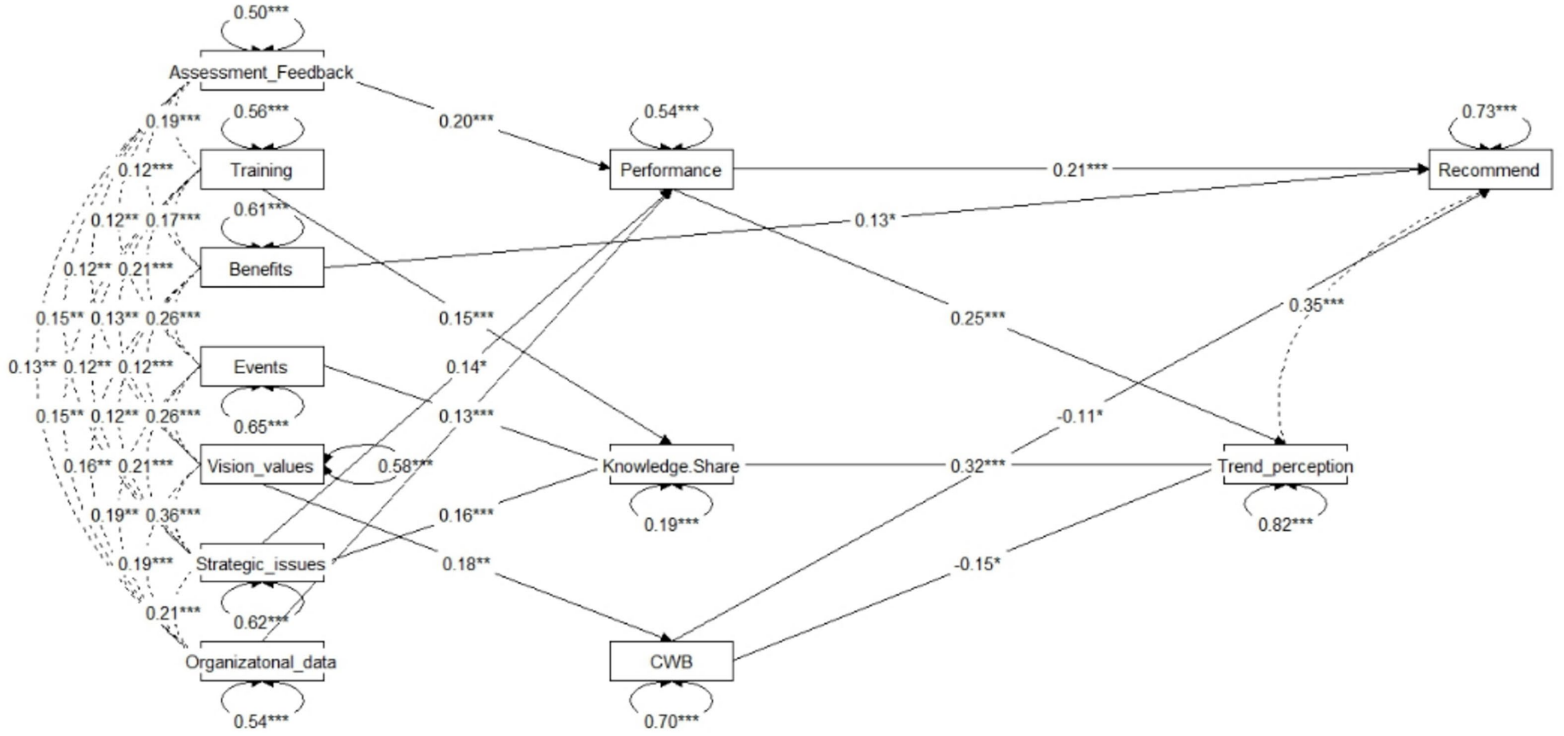
Path analysis coefficients.

Finally, to examine which specific HRM practices predict attitudes to freelancers willingness to recommend freelancers and trend in employing freelancers, a path analysis was conducted (See Table 5 and Figure 4). The final model showed a good fit (RMSEA = 0.06, CFI = 0.95, TLI = 0.90).

**Table 5:** Path analysis coefficients.

*IV*	*DV*	*b*	*se*	β	*p*	*95%CI*
Assessment/ Feedback	Performance	0.2	0.06	0.18	0.001	[0.08, 0.31]
Exposure to organizational data	Performance	−0.22	0.06	−0.21	<0.001	[−0.33, −0.1]
Exposure to strategic issues	Performance	0.14	0.06	0.14	0.01	[0.03, 0.25]
Training	Knowledge sharing	0.15	0.03	0.22	<0.001	[0.08, 0.22]
Participation in “team-spirit” Events	Knowledge sharing	0.13	0.03	0.20	<0.001	[0.06, 0.19]
Exposure to strategic issues	Knowledge sharing	0.16	0.03	0.25	<0.001	[0.09, 0.22]
Receiving Benefits	Engagement	0.15	0.04	0.20	<0.001	[0.07, 0.24]
Exposure to organizational vision	Engagement	−0.11	0.05	−0.14	0.04	[−0.21, 0.01]
Exposure to strategic issues	Engagement	0.13	0.05	0.16	0.01	[0.03, 0.23]
Exposure to organizational vision	CWB	0.18	0.06	0.16	<0.001	[0.07, 0.3]
Performance	Trend perception	0.25	0.06	0.20	<0.001	[0.13, 0.38]
Knowledge share	Trend perception	0.32	0.09	0.17	<0.001	[0.15, 0.49]
CWB	Trend perception	−0.15	0.06	−0.13	0.01	[−0.26, −0.04]
Performance	Freelancers’ recommendation	0.21	0.06	0.18	<0.001	[0.09, 0.33]
CWB	Freelancers’ recommendation	−0.11	0.05	−0.11	0.04	[−0.22, 0.001]
Receiving benefits	Freelancers’ recommendation	0.13	0.05	0.11	0.02	[0.09, 0.33]

*Only significant paths appears in this table.

The analysis revealed several significant mediation pathways. For Freelancers’ trend perception, the HRM practices of assessment and feedback had a positive indirect effect through performance (*β = 0.05, p = 0.01, 95% CI [0.01, 0.09]*), while exposure to organizational data had a negative indirect effect through performance (*β = −0.04, p = 0.01, 95% CI [−0.09, −0.01]*). Similarly, receiving training and instruction (*β = 0.04, p < 0.001, 95% CI [0.01, 0.08]*), participation in “team-spirit” events (*β = 0.04, p = 0.01, 95% CI [0.01, 0.07]*), and exposure to strategic issues (*β = 0.04, p < 0.001, 95% CI [0.02, 0.08]*) all had positive indirect effects through knowledge-sharing. In contrast, exposure to organizational vision had a marginally significant negative indirect effect through CWB (*β = −0.02, p = 0.05, 95% CI [−0.05, 0.00]*). For Freelancers’ recommendation, Assessment and feedback demonstrated a positive indirect effect via Performance (*β = 0.03, p = 0.02, 95% CI [0.01, 0.08]*), while Exposure to organizational data showed a negative indirect effect through Performance (*β = −0.04, p = 0.01, 95% CI [−0.08, −0.01]*). Finally, the indirect effect of Exposure to organizational vision on Freelancers’ recommendation through CWB was not significant (*β = −0.02, p = 0.09, 95% CI [−0.04, 0]*).

In summary, the results provide clear support for the two primary hypotheses concerning instrumental mechanisms. Both knowledge sharing and performance emerged as the central pathways linking HR practices to managerial perceptions of freelancer employment. In contrast, the additional relational pathways examined on an exploratory basis yielded limited or inconsistent support (i.e., engagement and OCB did not mediate the link between HR practices to managerial perceptions of freelancer employment, while CWB mediated this link).

## Discussion

This study examined how HRM practices or initiatives are associated with freelancers’ involvement or integration within the company are linked to specific behaviors, such as perceived performance and knowledge sharing, and how they mediate between HRM practices and managers’ perceptions of freelancers’ employment. The thorough analysis revealed the precise behaviors that mediate each practice to foster a favorable and desired attitude among managers regarding the employment of freelancers. The mediation disclosed will be the focus of this section. Consistent with the exploratory nature of this study, the findings provide selective support for the proposed mechanisms rather than uniform confirmation. Specifically, the results indicate that instrumental mechanisms (e.g., performance and knowledge sharing), which align with contractual and task-based exchanges, are more consistently supported. In contrast, relational mechanisms (e.g., engagement and OCB), which depend on identification and ongoing reciprocal relationships, appear more limited or conditional in freelance contexts. These findings suggest that HRM models developed in traditional employment settings do not fully generalize to freelance work and probably operate through a more constrained set of moderators. The discussion therefore focuses on distinguishing between mechanisms that appear more robust and those that are more contingent in freelance settings. By identifying which mechanisms operate more robustly in freelance settings, this study refines rather than fully confirms existing HRM and SET-based models. Accordingly, the findings should be interpreted as indicative patterns based on perceived behaviors rather than definitive evidence of objective behavioral effect. More specifically, the study demonstrates that performance and knowledge sharing are the primary mechanisms through which HR practices influence managerial perceptions of freelancers, whereas relational mechanisms appear limited in this context.

### Knowledge-sharing as a core instrumental mechanism

H1 was supported: knowledge-sharing mediated the relationship between HR practices and perceptions of freelancers. Training, exposure to strategic issues, and participation in social events all encouraged knowledge-sharing. Training provides resources that freelancers reciprocate by sharing expertise (Allen et al., 2016; Blau, 1964). Similarly, access to strategic information signals trust and enhances motivation to share knowledge (Kankanhalli et al., 2005; Wang and Noe, 2010). Informal social gatherings also foster trust and break down barriers, leading freelancers to contribute knowledge more freely (Gala et al., 2024).

### Performance as a core instrumental mechanism

H2 was confirmed: performance mediated the effects of assessment/feedback, exposure to strategy, and access to organizational data on managerial perceptions. Formal feedback, often absent for freelancers, was shown to enhance alignment and performance (Baird et al., 2020; Cappelli and Conyon, 2018). Similarly, exposure to strategic issues improved decision-making and adaptability (Wu et al., 2015; Kaplan and Norton, 2009), while access to organizational data enabled freelancers to anticipate challenges and perform more effectively (Deng et al., 2023).

Additional relational pathways examined on an exploratory basis:

*Engagement as an exploratory relational pathway.* Although engagement was examined as an additional relational pathway linking HRM practices to perceptions of freelancers’ employment, no mediation effect was found. Several practices (exposure to strategy, organizational vision, and benefits) were related to engagement, but this pathway did not significantly mediate managerial perceptions. This may be because engagement is closely tied to belonging and identification with the organization, which freelancers often lack (Koriat and Gelbard, 2014, 2019). They tend to prioritize professional expertise over organizational attachment. Still, as Zadik et al. (2019) argue, organizations increasingly expect freelancers to collaborate with permanent staff, suggesting a growing tension between transactional contracts and relational expectations (Rabenu, 2021).
*OCB as an exploratory relational pathway.* Although OCB was examined as an additional relational pathway between HRM practices and perceptions of freelancers, no significant associations were found. OCB typically emerges from social exchange when employees feel invested in by their employer (Chernyak-Hai and Rabenu, 2018; Organ, 2018). Freelancers, however, operate under narrowly defined contracts, which discourage discretionary behaviors beyond agreed deliverables. Organizations may also avoid encouraging OCB due to concerns about added costs or obligations, limiting the likelihood of freelancers engaging in such behaviors.
*CWB as an unexpected exploratory pathway.* Unexpectedly, CWB emerged as an additional exploratory pathway, whereby exposure to organizational vision and values was positively associated with CWB, which in turn reduced positive perceptions of freelancers. One possible interpretation is that exposure to organizational values may create cognitive dissonance for freelancers, especially when organizational rhetoric clashes with their independent identity or actual practices. Such misalignment can foster cynicism and lead freelancers to express dissatisfaction through counterproductive behaviors, reflecting a perceived breach of psychological contract. These findings should be interpreted with caution and warrant further investigation to establish their robustness and underlying mechanisms. A further explanation lies in an expectation misalignment: when organizations exposing freelancers to organizational values and vision, these are relational cues that may generate tension or cognitive dissonance rather than identification since the freelancers expectations are of instrumental mechanism (economic exchange). This discrepancy may result in adverse outcomes for both the individual and the organization (Tsui et al., 1997).

Looking at the research model (see Figure 2), the results fit with the expectations for instrumental exchange between the freelancers and the organization. Specifically, exchange theory conceptualizes economic terms of costs and rewards, which Suitable for freelancer–organization relationship. However, SET (Social Exchange Theory) which engagement and OCB can serve as its representative variables, extends this perspective by emphasizing reciprocity, which typically develops through ongoing interactions (Chernyak-Hai and Rabenu, 2018; Rabenu, 2021). The current research finds that the mediation effect of instrumental mechanisms in the relationship between HR practices applied to freelancers and the perceptions of hiring freelancers, appears to be the dominant mechanism. Relational mechanisms (social–emotional exchange) in the context of freelancers are not supported in the general context of freelancers. Accordingly, socio-emotional outcomes such as OCB, do not appear to represent the dominant mechanisms. Only specific types of freelancers, such as Semi-freelancers which are: “…freelancers (outsiders) that work with the organization for the long term” (Rabenu, 2021, p. 248), and therefore behave more similarly to employees, might fit the traditional SET rules. Thus, it should not be assumed that all freelancers respond uniformly to HR practices or to SET norms. A limitation of the present study is that it does not differentiate between freelancers who meet long term condition and those who do not. This possible explanation should be studied in further research.

### Theoretical contributions

This study contributes by moving beyond the assumption that HRM mechanisms derived from traditional employment relationships can be transferred intact to freelance work. Instead, we argue that in freelance contexts, HR practices are more likely to shape managerial perceptions through instrumental mechanisms closely tied to project execution and coordination, particularly performance and knowledge sharing. A central theoretical contribution of this study is in challenging the long-standing periphery distinction in HRM models (e.g., Lepak and Snell, 1999; Atkinson, 1984). These frameworks advise prioritizing core employees while limiting investment in freelancers, yet this view no longer reflects workforce realities. Freelancers are now engaged in strategic, knowledge-intensive projects central to competitiveness (Burke, 2015; Miller and Miller, 2012). Our findings show that when practices such as training, feedback, and access to strategic information are applied, managers evaluate freelancers more positively, underscoring the need to rethink HRM theory and position freelancers as a critical workforce segment.

A second contribution concerns Social Exchange Theory (SET) (Blau, 1964; Cropanzano and Mitchell, 2005). SET traditionally explains how employees reciprocate organizational investments with behaviors such as OCB, engagement, and knowledge-sharing. Freelancers, however, operate mainly through contractual, economic exchanges, leading to the assumption they would be unresponsive to HR practices. Our findings complicate this view: freelancers did reciprocate, but in nuanced ways; training and social inclusion fostered knowledge-sharing, and feedback enhanced performance, while exposure to organizational values unexpectedly increased counterproductive behavior. Thus, SET must be adapted to reflect freelancers’ contingent status, psychological distance, and unique interpretations of organizational practices. At the same time, our findings indicate that traditional SET mechanisms operate differently in freelance arrangements. Engagement and OCB, typically central relational pathways in employee settings, did not mediate the effects of HR practices on freelancers’ perceptions, and exposure to organizational values was associated with higher CWB. These patterns suggest that freelancers respond to HR inputs primarily through instrumental, task-related channels such as performance alignment and knowledge sharing, rather than through socio-emotional reciprocity. This reinforces the notion that freelancers hold narrower, more transactional psychological contracts, in which relational cues may generate tension or cognitive dissonance rather than identification. Accordingly, SET may require refinement when applied to independent professionals, emphasizing contingent, task-specific exchanges over enduring relational obligations.

A third contribution expands HRM research on non-standard work. Prior studies emphasize agency workers, part-time employees, or gig workers (De Stefano, 2016), but freelancers, especially highly skilled professionals in project-based roles, remain understudied. Our findings address this gap by treating freelancers as a distinct category: unlike gig workers, they negotiate contracts directly and collaborate on complex projects, yet remain external workers. By showing how HR practices shape their behaviors and managerial perceptions, we highlight that freelancers differ from other contingent workers and call for HR theories that recognize heterogeneity within non-standard employment. This distinction raises an important theoretical question regarding the boundary conditions of traditional HRM theories, such as Social Exchange Theory, when applied to freelance work arrangements. By explicitly situating the model within freelancers’ multi-client, ecosystem-based careers, we contribute to emerging scholarship on inclusive HRM and demonstrate how selective HR practices function within complex, boundary-spanning employment arrangements.

Finally, our findings contribute to psychological contract theory. Psychological contracts describe individuals’ beliefs about reciprocal obligations in the employment relationship (Rousseau, 1995). For employees, breaches of these expectations are linked to reduced commitment, lower performance, and increased CWB (Zhao et al., 2007). For freelancers, however, psychological contracts are more complex. Such as when exposure to organizational values highlights contradictions in the exchange expectations (since they are relational cues for exchange rather than instrumental), freelancers may respond with counterproductive behaviors. Thus, psychological contracts also apply to freelancers, but they are fragile and shaped by the tension between transactional and relational elements.

### Practical implications

The findings of this study provide several implications for organizations managing a growing freelance workforce. Freelancers are not employees, yet they share many of the same human needs: to belong, to be recognized, and to build positive relationships. Our results suggest that when organizations extend support through training, feedback, and limited social inclusion, freelancers perform better, share knowledge more readily, and contribute more conscientiously. Treating freelancers as valued contributors, rather than transactional contractors, can therefore yield organizational benefits.

Managers working directly with freelancers should also consult HR professionals to determine which practices can maximize engagement and minimize risks. Sometimes HR practices yield surprising, even counterintuitive effects—as in our finding regarding exposure to organizational values and CWB. Managers should therefore attend to the mechanisms underlying exchanges with freelancers, because these mechanisms (relational, instrumental) are critical for aligning expectations between freelancers and the organization.

Finally, given the continued global growth of freelancing, policymakers should consider updating labor legislation to enable organizations to engage freelancers more effectively without undermining their independent status. Clearer legal frameworks could help both organizations and freelancers benefit from a more balanced, transparent, and productive relationship.

### Limitations and future research

This study has several limitations. First, we relied on newly developed measures, which may limit the depth and breadth of construct operationalization. While the scales were statistically validated, future research should employ established instruments to strengthen comparability with prior work. In addition, the dependent variable is based on two items capturing general perceptions toward freelancer employment. Although such concise measures have been shown to be valid for capturing overall attitudes, they may not fully reflect the multidimensional nature of organizational decision-making. Several constructs were also measured using perception-based and indirect indicators, including comparative evaluations of freelancers relative to salaried employees and aggregated indices derived from categorical response formats. While this approach aligns with our focus on managerial perceptions, it may introduce subjectivity and limit the precision of behavioral measurement. As such, the findings should be interpreted as reflecting perceived rather than actual behaviors, which may be subject to bias or contextual interpretation. Future research should incorporate multi-source data, including freelancers’ self-reports, supervisor ratings, or objective performance indicators, as well as validated continuous scales to strengthen construct validity. Second, participants were drawn from organizations that contract freelancers, yet many may not have directly managed or supervised them, limiting the depth of their insights. Future studies should focus on managers with firsthand experience and also include freelancers’ own perspectives, which are absent from the current study.

Third, as HRM research on freelancers is still in its early stages, qualitative approaches could provide richer accounts of their experiences and complement quantitative findings. Future research may also benefit from testing more parsimonious models that focus on the most robust mechanisms identified in this study. Moreover, the present study does not differentiate between freelancers who work for long term in the organization and those who do not. The long/short term of contract may behave as moderator in the relationship between HR practices and freelancers’ behavior. This should be studied in further research to better refine SET models for freelancers.

Fourth, our study defined freelancers narrowly as self-employed professionals working under project-based contracts. Broader definitions, including platform or gig workers, as well as comparisons across industries, would allow for a more comprehensive understanding of differences within non-standard work arrangements. Future research could also examine how freelancers’ simultaneous relationships with multiple organizations shape their responses to HR practices across client portfolios.

Finally, although our dependent variables reflect managerial perceptions rather than direct behavioral or organizational outcome measures, one of them, willingness to recommend hiring freelancers, captures a behavioral intention that is closely tied to actual staffing decisions (Subramony, 2011). Understanding these evaluative intentions is an important first step, as prior work demonstrates that connection between attitudes and behaviors at work in general (Kammeyer-Mueller et al., 2024) and for recruiting freelancers in particular (Shwartz-Asher et al., 2025). At the same time, decisions regarding the engagement of freelancers are often embedded within broader organizational constraints, including budgetary considerations, procurement procedures, and workforce planning strategies. Accordingly, managerial perceptions should be understood as one important input in these decisions rather than the sole determinant of organizational hiring practices. Future studies should focus on developing a broader model to identify the various variables that influence freelancer hiring decisions. Nevertheless, future research could extend the model by examining more consequential indicators such as actual re-engagement decisions, project-level performance outcomes, or broader organizational implications of freelancer integration.

## Conclusion

Freelancers are no longer peripheral actors but central contributors to organizational performance, particularly in knowledge-intensive and innovative projects. However, HR theory and practice have been slow to account for their unique role. Overall, the findings suggest that selected HRM practices are associated with more positive managerial perceptions of freelancers, primarily through perceived performance and knowledge-sharing pathways. By extending HRM theory to freelancers, we move beyond a narrow focus on core employees and acknowledge the diverse ways organizations engage talent. These findings should be interpreted as reflecting managerial evaluations and perceived mechanisms rather than directly observed freelancer behaviors, and future research should further examine these relationships using multi-source and behavioral data.

## Data Availability

The raw data supporting the conclusions of this article will be made available by the authors, without undue reservation.

## References

[ref1] Adams A. Freedman J. Prassl J. (2018). Rethinking legal taxonomies for the gig economy. Oxf. Rev. Econ. Policy 34, 475–494. doi: 10.1093/oxrep/gry006

[ref2] Akhmetshin E. M. Kovalenko K. E. Mueller J. E. Khakimov A. K. Yumashev A. V. Khairullina A. D. (2018). Freelancing as a type of entrepreneurship: advantages, disadvantages and development prospects. J. Entrep. Educ. 21, 1528–2651.

[ref3] Al Haraisa Y. E. (2022). The impact of strategic alignment and strategic awareness on strategic performance: evidence from Jordan. Int. J. Acad. Res. Account. Finan. Manage. Sci. 12, 42–55. doi: 10.6007/IJARAFMS/v12-i4/15676

[ref4] Allen T. J. Gloor P. Fronzetti Colladon A. Woerner S. L. Raz O. (2016). The power of reciprocal knowledge sharing relationships for startup success. J. Small Bus. Enterp. Dev. 23, 636–651. doi: 10.1108/JSBED-08-2015-0110

[ref5] Ang L. Eisend M. (2018). Single versus multiple measurement of attitudes: a meta-analysis of advertising studies validates the single-item measure approach. J. Advert. Res. 58, 218–227. doi: 10.2501/JAR-2017-001

[ref6] Atkinson J. (1984). Manpower strategies for flexible organizations. Pers. Manag. 16, 28–31.

[ref7] Baird K. Tung A. Su S. (2020). Employee empowerment, performance appraisal quality and performance. J. Manag. Control. 31, 451–474. doi: 10.1007/s00187-020-00307-y

[ref8] Barlage M. van den Born A. van Witteloostuijn A. (2023). The needs of freelancers and the characteristics of “gigs”: creating beneficial relations between freelancers and their hiring organizations. Emer. Open Res. 1, 1–23. doi: 10.12688/emeraldopenres.12928.1

[ref9] Barney J. B. (2018). Why resource-based theory's model of profit appropriation must incorporate a stakeholder perspective. Strat. Manage. J. 39, 3305–3325. doi: 10.1002/smj.2949

[ref10] Beer M. Boselie P. Brewster C. (2015). Back to the future: implications for the field of HRM of the multistakeholder perspective proposed 30 years ago. Hum. Resour. Manag. 54, 427–438. doi: 10.1002/hrm.21726

[ref11] Benner M. J. Tushman M. L. (2003). Exploitation, exploration, and process management: the productivity dilemma revisited. Acad. Manag. Rev. 28, 238–256. doi: 10.5465/amr.2003.9416096

[ref12] Bergkvist L. Rossiter J. R. (2007). The predictive validity of multiple-item versus single-item measures of the same constructs. J. Mark. Res. 44, 175–184. doi: 10.1509/jmkr.44.2.175

[ref13] Blau P. M. (1964). Exchange and power in social life. New York: Wiley.

[ref14] Boxall P. Purcell J. (2022). Strategy and human Resource Management. Bloomsbury Publishing.

[ref15] Burke A. (2015). Introduction: a freelancing and self-employment research agenda. Int. Rev. Entrepreneursh. 13, 1–6.

[ref16] Butterick M. Charlwood A. (2021). HRM and the COVID-19 pandemic: how can we stop making a bad situation worse? Hum. Resour. Manage. J. 31, 847–856. doi: 10.1111/1748-8583.12344

[ref17] Camuffo A. De Stefano F. (2019). “Getting access to strategic human capital resources: a multiple strategic factor market approach,” in Handbook of Research on Strategic human capital Resources, eds., D. G. Collings, K. Mellahi, and W. F. C ascio (Cheltenham, UK: Edward Elgar Publishing), 281–306.

[ref18] Capatina A. Juarez-Varon D. Micu A. Micu A. E. (2024). Leveling up in corporate training: unveiling the power of gamification to enhance knowledge retention, knowledge sharing, and job performance. J. Innov. Knowl. 9, 25–51. doi: 10.1016/j.jik.2024.100530

[ref19] Cappelli P. Conyon M. J. (2018). What do performance appraisals do? ILR Rev. 71, 88–116. doi: 10.1177/0019793917698649

[ref20] Carpenter N. C. Whitman D. S. Amrhein R. (2021). Unit-level counterproductive work behavior (CWB): a conceptual review and quantitative summary. J. Manage. 47, 1498–1527. doi: 10.1177/0149206320978812

[ref21] Chang S.-J. van Witteloostuijn A. Eden L. (2010). From the editors: common method variance in international business research. J. Int. Bus. Stud. 41, 178–184. doi: 10.1057/jibs.2009.88

[ref22] Chernyak-Hai L. Rabenu E. (2018). The new era workplace relationships: is social exchange theory still relevant? Ind. Organ. Psychol. 11, 456–481. doi: 10.1017/iop.2018.5

[ref23] Cropanzano R. Mitchell M. S. (2005). Social exchange theory: an interdisciplinary review. J. Manage. 31, 874–900. doi: 10.1177/0149206305279602

[ref24] Cross D. Swart J. (2022). The (ir) relevance of human resource management in independent work: challenging assumptions. Hum. Resour. Manage. J. 32, 232–246. doi: 10.1111/1748-8583.12389

[ref9003] De Stefano V. (2016). Introduction: crowdsourcing, the gig-economy and the law.. Comparative Labor Law & Policy Journal, 37, 1–10.

[ref26] Deng H. Duan S. X. Wibowo S. (2023). Digital technology driven knowledge sharing for job performance. J. Knowl. Manag. 27, 404–425. doi: 10.1108/JKM-08-2021-0637

[ref25] De Vos A. Van der Heijden B. I. (2017). Current thinking on contemporary careers:the key roles of sustainable HRM and sustainability of careers. Curr. Opin. Environ. Sustain. 28, 41–50. doi: 10.1016/j.cosust.2017.07.003

[ref27] Fong C. Y. Ooi K. B. Tan B. I. Lee V. H. Chong A. Y. L. (2011). HRM practices and knowledge sharing: an empirical study. Int. J. Manpow. 32, 704–723. doi: 10.1108/01437721111158288

[ref28] Gala B. Reyes V. Seifi L. Lamba M. (2024). Relationship building through informal gatherings and technology integrations: a case study. Libr. Hi Tech News 41, 25–28. doi: 10.1108/LHTN-12-2023-0219

[ref29] Gong T. (2025). Algorithmic management and gig workers: engagement, exhaustion and citizenship behavior. Manage. Decis. 2025, 1–22. doi: 10.1108/MD-01-2025-0111

[ref30] Griep Y. Vantilborgh T. Baillien E. Pepermans R. (2016). The mitigating role of leader–member exchange when perceiving psychological contract violation: a diary survey study among volunteers. Eur. J. Work Organ. Psychol. 25, 254–271. doi: 10.1080/1359432X.2015.1046048

[ref31] Griffin R. W. O’Leary-Kelly A. M. (2004). “An introduction to the dark side,” in The Dark Side of Organizational Behavior, eds. R. W. Griffin and A. M. O’Leary-Kelly (San Francisco, CA: Jossey-Bass), 1–19.

[ref32] Jacobs S. De Vos A. Stuer D. Van der Heijden B. I. (2019). “Knowing me, knowing you” the importance of networking for freelancers’ careers: examining the mediating role of need for relatedness fulfillment and employability-enhancing competencies. Front. Psychol. 10, 1–14. doi: 10.3389/fpsyg.2019.02055, 31572262 PMC6751263

[ref33] Kammeyer-Mueller J. D. Rubenstein A. L. Barnes T. S. (2024). The role of attitudes in work behavior. Annu. Rev. Organ. Psychol. Organ. Behav. 11, 221–250. doi: 10.1146/annurev-orgpsych-101022-101333

[ref34] Kankanhalli A. Tan B. C. Wei K. K. (2005). Contributing knowledge to electronic knowledge repositories: an empirical investigation. MIS Q. 2005, 113–143. doi: 10.2307/25148670

[ref35] Kaplan R. S. Norton D. P. (2009). “Putting the balanced scorecard to work,” in The economic impact of Knowledge, eds. D. W. Jorgenson and K. M. Vu (London, UK: Routledge), 315–324.

[ref36] Kitching J. Smallbone D. (2012). Are freelancers a neglected form of small business? J. Small Bus. Enterprise Dev. 19, 74–91. doi: 10.1108/14626001211196415

[ref37] Koriat N. Gelbard R. (2014). Knowledge sharing motivation among IT personnel: integrated model and implications of employment contracts. Int. J. Inf. Manag. 34, 577–591. doi: 10.1016/j.ijinfomgt.2014.04.009

[ref38] Koriat N. Gelbard R. (2019). Knowledge sharing analytics: the case of IT workers. J. Comput. Inf. Syst. 59, 308–318. doi: 10.1080/08874417.2017.1360163

[ref39] Leighton P. Wynn M. (2011). Classifying employment relationships--more sliding doors or a better regulatory framework? Ind. Law J. 40, 5–44. doi: 10.1093/indlaw/dwq029

[ref40] Lepak D. P. Snell S. A. (1999). The human resource architecture: toward a theory of human capital allocation and development. Acad. Manag. Rev. 24, 31–48. doi: 10.5465/amr.1999.1580439

[ref41] Lo Presti A. Pluviano S. Briscoe J. P. (2018). Are freelancers a breed apart? The role of protean and boundaryless career attitudes in employability and career success. Hum. Resour. Manag. J. 28, 427–442. doi: 10.1111/1748-8583.12188

[ref42] McKeown T. Pichault F. (2021). Independent professionals as talent: evidence from individual views of working as a contractor. Hum. Resour. Manag. 60, 313–328. doi: 10.1002/hrm.22045

[ref43] Memon M. A. Salleh R. Mirza M. Z. Cheah J. H. Ting H. Ahmad M. S. . (2021). Satisfaction matters: the relationships between HRM practices, work engagement and turnover intention. Int. J. Manpow. 42, 21–50. doi: 10.1108/IJM-04-2018-0127

[ref44] Miller J. G. Miller M. (2012). The rise of the supertemp. Harv. Bus. Rev. 90, 50–62.

[ref45] OECD (2021) Self-Employment Rate (Indicator). Paris, France: Organisation for Economic Co-operation and Development. 10.1787/fb58715e-en

[ref46] ONS (2018) *Trends in Self-Employment in the UK*. Available online at: https://www.ons.gov.uk/employmentandlabourmarket/peopleinwork/employmentandemployeetypes/articles/trendsinselfemploymentintheuk/2018-02-07 (Accessed February 20, 2018).

[ref47] Organ D. W. (2018). Organizational citizenship behavior: recent trends and developments. Annu. Rev. Organ. Psychol. Organ. Behav. 5, 295–306. doi: 10.1146/annurev-orgpsych-032117-104536

[ref48] Poon T. S. C. (2019). Independent workers: growth trends, categories, and employee relations implications in the emerging gig economy. Employ. Responsib. Rights J. 31, 63–69. doi: 10.1007/s10672-018-9318-8

[ref49] Rabenu E. (2021). Twenty-First Century Workplace Challenges: Perspectives and Implications for Relationships in New Era Organizations. Lanham, MD: Rowman & Littlefield.

[ref9001] Rousseau D. (1995). Psychological contracts in organizations: Understanding written and unwritten agreements. Sage.

[ref9002] Santra S. (2021). Contingent workforce management: a holistic overview. Strategic HR Review, 20, 199–205. doi: 10.1108/SHR-08-2021-0035

[ref50] Shwartz-Asher D. Tziner A. Kurlander Y. Rabenu E. Shertzer Y. Ehrlich S. . (2025). Attitudes in action: how managers perceive and position freelancers in the modern workplace. J. Work Organ. Psychol. 41, 103–110. doi: 10.5093/jwop2025a11

[ref51] Snider L. (2018). Enabling exploitation: law in the gig economy. Crit. Criminol. 26, 563–577. doi: 10.1007/s10612-018-9416-9

[ref52] Spreitzer G. M. Cameron L. Garrett L. (2017). Alternative work arrangements: two images of the new world of work. Annu. Rev. Organ. Psychol. Organ. Behav. 4, 473–499. doi: 10.1146/annurev-orgpsych-032516-113332

[ref53] Spurk D. Straub C. (2020). Flexible employment relationships and careers in times of the COVID-19 pandemic. J. Vocat. Behav. 119, 103435–103435. doi: 10.1016/j.jvb.2020.103435, 32382161 PMC7204672

[ref54] Stirpe L. Profili S. Sammarra A. (2022). Satisfaction with HR practices and employee performance: a moderated mediation model of engagement and health. Eur. Manag. J. 40, 295–305. doi: 10.1016/j.emj.2021.06.003

[ref55] Subramony M. (2011). Antecedents and outcomes of contingent workers' attitudes toward their temporary help services firm: a unit level longitudinal investigation. J. Organ. Behav. 32, 850–868. doi: 10.1002/job.716

[ref56] Taamneh A. Alsaad A. K. Elrehail H. (2018). HRM practices and the multifaceted nature of organization performance: the mediation effect of organizational citizenship behavior. EuroMed J. Bus. 13, 315–334. doi: 10.1108/EMJB-02-2018-0010

[ref57] Topa G. Aranda-Carmena M. De-Maria B. (2022). Psychological contract breach and outcomes: a systematic review of reviews. Int. J. Environ. Res. Public Health 19, 15–27. doi: 10.3390/ijerph192315527, 36497602 PMC9737235

[ref58] Tsui A. S. Pearce J. L. Porter L. W. Tripoli A. M. (1997). Alternative approaches tothe employee organization relationship: does investment in employees pay off? Acad. Manag. J. 40, 1089–1121. doi: 10.2307/256928

[ref59] van den Groenendaal S. M. E. Freese C. Poell R. F. Kooij D. T. (2023). Inclusive human resource management in freelancers' employment relationships: the role of organizational needs and freelancers' psychological contracts. Hum. Resour. Manag. J. 33, 224–240. doi: 10.1111/1748-8583.12432

[ref60] Van Noordwijk M. Villamor G. B. Hofstede G. J. Speelman E. N. (2023). Relational versus instrumental perspectives on values of nature and resource management decisions. Curr. Opin. Environ. Sustain. 65, 1–9. doi: 10.1016/j.cosust.2023.101374

[ref61] Vučeković M. Avlijaš G. Marković M. R. Radulović D. Dragojević A. Marković D. (2023). The relationship between working in the “gig” economy and perceived subjective well-being in Western Balkan countries. Front. Psychol. 14, 1–10. doi: 10.3389/fpsyg.2023.1180532, 37377706 PMC10291236

[ref62] Wang S. Noe R. A. (2010). Knowledge sharing: a review and directions for future research. Hum. Resour. Manag. Rev. 20, 115–131. doi: 10.1016/j.hrmr.2009.10.001

[ref63] Wu S. P. J. Straub D. W. Liang T. P. (2015). How information technology governance mechanisms and strategic alignment influence organizational performance. MIS Q. 39, 497–518. doi: 10.2307/26628363

[ref64] Zadik Y. Bareket-Bojmel L. Tziner A. Shloker O. (2019). Freelancers: a manager’s perspective on the phenomenon. Rev. Psicol. Trab. Organ. 35, 39–48. doi: 10.5093/jwop2019a5

[ref65] Zhao H. A. O. Wayne S. J. Glibkowski B. C. Bravo J. (2007). The impact of psychological contract breach on work-related outcomes: a meta-analysis. Pers. Psychol. 60, 647–680. doi: 10.1111/j.1744-6570.2007.00087.x

